# Mammalian DNA Polymerase Kappa Activity and Specificity

**DOI:** 10.3390/molecules24152805

**Published:** 2019-08-01

**Authors:** Hannah R. Stern, Jana Sefcikova, Victoria E. Chaparro, Penny J. Beuning

**Affiliations:** Department of Chemistry & Chemical Biology, Northeastern University, Boston, MA 02115, USA

**Keywords:** Y-family, DNA replication, DNA damage, translesion DNA synthesis, primer extension, chemotherapy resistance

## Abstract

DNA polymerase (pol) kappa is a Y-family translesion DNA polymerase conserved throughout all domains of life. Pol kappa is special6 ized for the ability to copy DNA containing minor groove DNA adducts, especially *N*^2^-dG adducts, as well as to extend primer termini containing DNA damage or mismatched base pairs. Pol kappa generally cannot copy DNA containing major groove modifications or UV-induced photoproducts. Pol kappa can also copy structured or non-B-form DNA, such as microsatellite DNA, common fragile sites, and DNA containing G quadruplexes. Thus, pol kappa has roles both in maintaining and compromising genomic integrity. The expression of pol kappa is altered in several different cancer types, which can lead to genome instability. In addition, many cancer-associated single-nucleotide polymorphisms have been reported in the *POLK* gene, some of which are associated with poor survival and altered chemotherapy response. Because of this, identifying inhibitors of pol kappa is an active area of research. This review will address these activities of pol kappa, with a focus on lesion bypass and cellular mutagenesis.

## 1. Introduction

**Figure 1:**
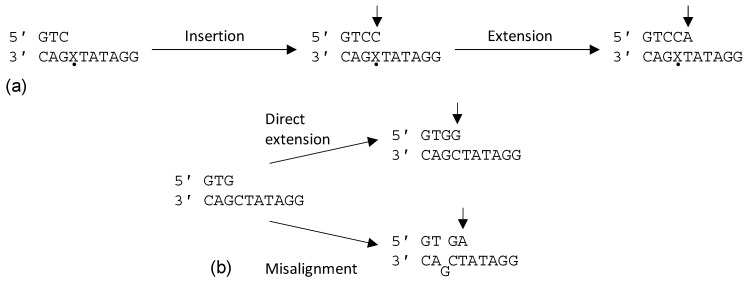
(**a**) Primer extension by translesion synthesis showing both the insertion and extension steps. The dot indicates the site of a lesion. (**b**) Direct extension (top) versus a one-nucleotide deletion resulting from looping a template base out of the DNA helix.

DNA is under constant threat of damage from both external sources, such as UV light and environmental pollution, and internal sources, such as reactive oxygen species. Cells have several responses to process the damage, such as repairing or bypassing the damage. Bypassing damage by copying damaged DNA is the mechanism of Y-family DNA polymerases (pols) in a process known as translesion synthesis (TLS) [1]. Y-family DNA polymerases insert nucleotides opposite damaged bases, and then the same or a different Y-family polymerase extends the new primer terminus past the damage in the extension step of TLS (Figure 1) [1]. Y-family DNA polymerases have larger, more accommodating active sites than replicative DNA polymerases [2,3], though they are less processive than replicative polymerases; for example, human pol kappa (κ) incorporates ~20–30 nucleotides per binding event [4]. Y-family DNA polymerases are found in all domains of life and are specialized to bypass particular types of damage. Mammals have four Y-family DNA polymerases: pol η, pol ι, pol κ, and Rev1 [4]. Pol κ differs from the other four mammalian Y-family pols in that its orthologs exist in bacteria and archaea [5,6,7,8]. Pol κ, like its *Escherichia coli* counterpart DinB, processes DNA adducts mainly at the *N*^2^ position of guanine in an error-free manner [6,9,10,11,12,13,14,15,16,17]. Mammalian DNA polymerase κ will be the subject of this review.

## 2. Structure

**Figure 2:**
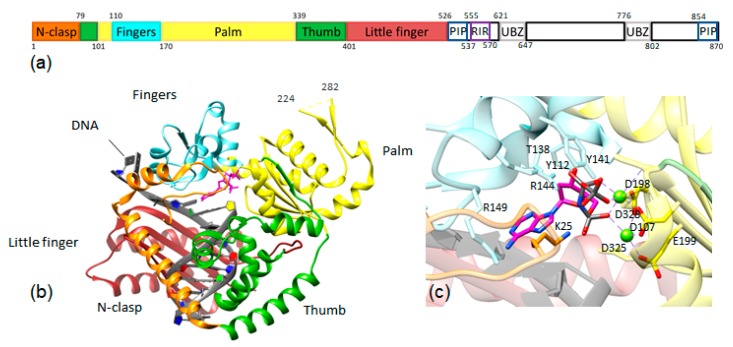
(**a**) Diagram of the domains of human pol κ: In addition to the polymerase domains, PCNA-interacting peptide (PIP) regions, Rev1-interacting region (RIR), and ubiquitin-binding zinc finger (UBZ) domains are shown. (**b**) The structure of the polymerase domain of human pol κ with domains colored as in Figure 2a (PDB ID: 6CST) [23]. (**c**) Close-up view of the structure of pol κ highlighting the active site residues in contact with the incoming nucleotide. The catalytic residues D107, D198, and E199 are shown in yellow sticks with red oxygen atoms; others are shown as sticks colored by the domain as in Figure 2a,b. Metal ions are shown as green spheres.

Y-family DNA polymerases are composed of the domains that replicative DNA polymerases also typically contain—the thumb, the palm, and the finger domains—but they also contain a fourth, unique domain called the little finger or polymerase-associated domain (PAD) that makes contacts with the major groove of DNA (Figure 2) [2,18,19,20]. DNA pol κ also has a fifth, unique domain at the N-terminus called the N-clasp [21]. The N-clasp has been observed to interact with DNA and to stabilize the ternary complex polymerase bound with DNA and deoxyribonucleotide triphosphate (dNTP) through contacts with the catalytic core, the little finger, and the DNA duplex region [22]. Although human DNA pol κ contains 870 amino acid residues, it was determined through primer extension assays using protein truncations that residues 19–526 are sufficient to maintain wild type-like polymerization activity [21]. It also has been observed that truncated constructs of the protein are more stable than the full-length protein [6] and, thus, are more commonly used in experiments. Removal of the N-clasp significantly reduces activity and, because of its interactions with the DNA, has implications for lesion bypass. Until recently, the crystal structures of pol κ lacked sufficient resolution to identify interactions between the N-clasp residues and other parts of the complex. However, a 2.0-Å resolution crystal structure was reported in 2018 that shows structural characteristics and interactions crucial for our understanding of the role of the N-clasp [23]. This crystal structure shows that the N-terminal residues are well-ordered and interact with the N-clasp, fingers, and thumb domains. The electron density around R18 and K25 is weaker than other residues, which the authors argue demonstrates high flexibility [23]. The active site contains many ordered water molecules engaged in an extensive network of hydrogen bonds, especially with the highly conserved residues R18, R149, and K25. K25 was identified as having a key role in activity through primer extension assays and kinetics assays. Pol κ K25 is at an analogous position and proposed to function similarly to a conserved lysine residue in the O-helix of pol I that makes contacts with phosphates on the incoming nucleotide [23]; pol I K758 is involved in dNTP binding through a conformational change in the fingers domain. However, while Y-family pols do not undergo a similar conformational change in the fingers domain as A-family DNA pols [24,25,26,27,28], this lysine residue K25 still acts as a proton donor to facilitate the formation of pyrophosphate and the reaction to form the phosphodiester bond [23].

## 3. Regulation

Mouse pol κ expression is regulated by p53 and UV or doxorubicin DNA damage, whereas human pol κ is not [29]. The promoter region of human pol κ harbors binding sites for stimulating protein-1 (SP1) and cyclic AMP-responsive element binding protein (CREB) that positively regulate pol κ expression [30]. Human pol κ expression is increased by exposure to benzo[a]-pyrene diol epoxide (BPDE) [31], and human, rat, and mouse pol κ expression is dependent on the aryl hydrocarbon receptor [32,33]. In addition, human pol κ is upregulated in lung cancers and gliomas, and in some cases this is correlated with genomic instability, while pol κ is downregulated in colorectal cancer [30,34,35,36,37].

In addition to two proliferating cell nuclear antigen (PCNA)-interacting peptides (PIP-boxes) and a Rev1-interacting region [38], pol κ contains two ubiquitin-binding zinc finger (UBZ) domains (Figure 2). The UBZ domains can bind monoubiquitylated PCNA, but upon binding ubiquitin, they undergo a conformational change to inhibit the interaction of Y-family polymerases with Ub-PCNA or other ubiquitylated proteins. This inhibition prevents the polymerase from targeting damaged DNA, so it can no longer participate in TLS [2]. Cells harboring a pol κ variant with a stronger p21-derived PIP box obviated the UBZ domain for pol κ-dependent genome instability [39]. Studies of mouse pol κ have revealed that the UBZ domains are required for pol κ to form nuclear foci after UV exposure [40], presumably reflecting their importance for recruitment of pol κ to sites of DNA replication.

## 4. Fidelity and Selectivity

Because Y-family pols lack an intrinsic proofreading domain and can incorporate nucleotides opposite damaged DNA bases that block high-fidelity pols, the Y-family pols are often associated with high error rates leading to increased mutagenesis [2]. However, pol κ is the most faithful of the Y-family pols with an error rate 10^−3^ to 10^−4^ on undamaged templates [4,41,42]. Studies of members of other DNA polymerase families crystallized in several stages of the catalytic cycle have revealed an important conformational change that serves as a fidelity checkpoint in which the fingers domain transitions from open to closed upon binding the dNTP [24]. The crystal structures of pol κ generally show a large conformational change upon binding DNA, but comparison of crystal structures of binary versus ternary structures are relatively similar regardless of dNTP identity [21,22]. However, kinetics, thermal stability assays, and hydrogen–deuterium exchange-mass spectrometry (HDXMS) experiments indicate that pol κ adopts a specific, active conformation only when the correct or preferred substrates are bound, i.e., undamaged DNA or *N*^2^-furfuryl-dG (minor groove damage)-containing templates and the correct incoming dNTP [43,44]. The palm domain in DNA polymerases is associated with catalysis [2]; however, regions in the palm, thumb, and little finger domain were observed to participate in this conformational change. These regions are distally located to the active site, and some do not make contact with the incoming dNTP [43]. This work not only demonstrates the importance of more distal regions of the protein and their ability to discriminate between the correct and incorrect incoming nucleotide but also provides evidence for a fidelity checkpoint in pol κ.

**Figure 3:**
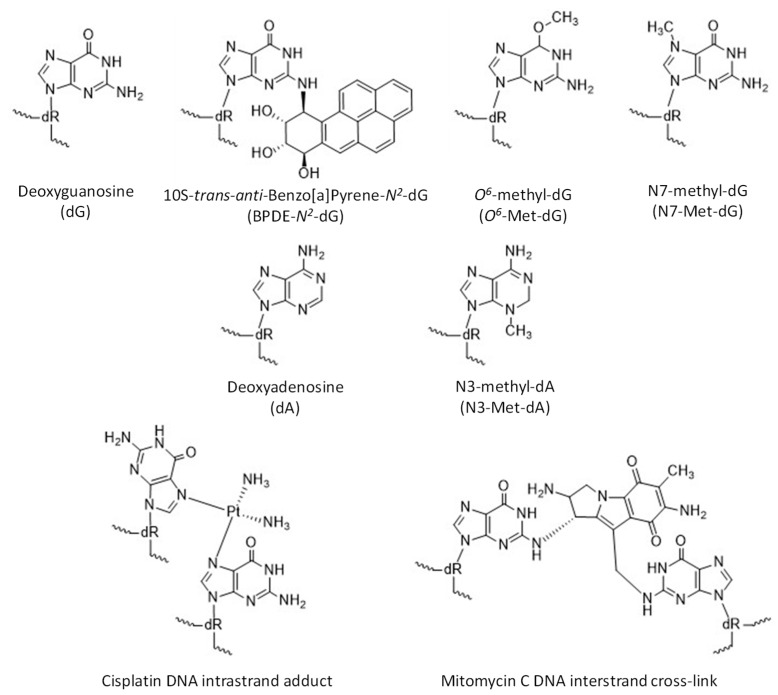
Structures of undamaged dG and dA and examples of damaged DNA bases.

Pol κ is observed to bypass several types of damage at the minor groove of DNA and, generally, is inhibited by major groove DNA adducts (Figure 3). Pol κ has been observed to bypass thymine glycol in an error-free manner [45] and etheno-deoxyadenosine, albeit poorly [46]. Pol κ can also copy DNA containing abasic sites by incorporating dA across from the lesion, though with low efficiency [17,47]. Pol κ is inhibited by the major groove adducts BPDE-*N^6^*-dA [14], *N*^6^-furfuryl-dA [48], and bulky *O*^6^-alkyl-dG modifications [49,50]. Pol κ can replicate DNA templates containing various bulky and non-bulky lesions, such as BPDE-*N^2^*-dG [10,14,16,17,51,52,53,54,55], *N*^2^-(1-carboxyethyl)-dG [56,57], 8-oxo-dG [17], *N*^2^-alkyl-dG [10,58], *O*^2^- and some *O*^4^-alkyl-dT adducts [59,60,61,62], thymine glycol [45], DNA-peptide crosslinks, and intrastrand adducts and interstrand crosslinks (ICL) formed between purine bases and cisplatin [6,10,17,63,64,65,66,67,68,69,70].

Bypass of 2-acetylaminofluorene-dG can be either error-free or error-prone [16,17,71]. Although pol κ is blocked by the UV-induced T-T cyclobutane pyrimidine dimer (CPD) and the (6-4) photoproduct lesions, the enzyme can extend past a dG inserted opposite the 3′T in the T-T dimer [72]. Pol κ is also inhibited by several major groove purine adducts, such as *N*^6^-furfuryl-dA and etheno-dA [48]. The use of isosteric base pairs that lack hydrogen bonding capabilities revealed that pol κ requires hydrogen bonding for efficient primer extension, unlike some replicative DNA polymerases [73]. The 3-aza-dG base analog, missing only the minor groove hydrogen bond acceptor N3, was replicated with reasonable efficiency by pol κ [73].

Examination of the structures of pol κ and comparison to sequences of other Y-family DNA polymerases have provided insights into pol κ preferences for certain lesions. Pol κ was shown to be tolerant to changes in its active site loop comprising residues 127–141 [74]. Notably, pol κ V130I had relaxed discrimination against the major groove adduct *N*^6^-furfuryl-dA, which is somewhat analogous to the DinB R35A mutation, indicating the importance of this region for lesion selectivity [74]. Within the same active site loop, pol κ M135 is suggested to be too bulky to accommodate T-T CPD lesions whereas human pol η, which can bypass T-T CPDs, has a glycine in a similar position; this could be a way to select DNA lesions so that the correct polymerase works on its associated adducts [9]. Similarly, analysis of inhibitor binding to pol κ suggests that the M135 and A151 side chains limit the orientation of the nascent base pair in the active site [75].

The ability to discriminate between ribonucleotides (rNTPs) and deoxyribonucleotides (dNTPs) is important to prevent ribonucleotide incorporation into the extending primer strand as this could lead to genomic instability [6,76]. Because rNTP concentrations are as much as 1000-fold higher in the cell than dNTP concentrations [77,78], polymerases must have a way to discriminate against rNTPs. Through the alignment of many Y-family pols, site-directed mutagenesis, and primer extension assays, the residue Y112 was determined to be the steric gate in pol κ as the alanine and valine variants both incorporated rNTPs [6]. However, these variants incorporate rNTPs less efficiently than dNTPs [6]. HDXMS experiments showed that pol κ adopts a substrate-specific conformational change when the correct substrate dNTP is bound versus the incorrect substrate rNTP [76]. The polymerase assumes a similar conformation when an rNTP is bound, but to a lesser extent, which can help to explain the decrease in activity when only rNTPs are present [76].

### 4.1. Role in TLS Extension Step

During TLS, a Y-family polymerase bypasses the damage and then either the same polymerase extends past the damage or another Y-family polymerase is recruited to extend past the damage so that a replicative polymerase can subsequently continue DNA synthesis [1,2]. Most eukaryotic TLS follows this two-step response, in which pol κ is generally thought to be specialized for the extension step, especially in extending mispaired primer termini [2,72,79,80,81]. While pol κ has a misincorporation frequency of 10^−3^ to 10^−4^ on undamaged templates [41], it also extends from mispaired primer termini with a greater proficiency at about 10^−1^ to 10^−2^ [72,81]. It has been suggested that mispaired primer termini are preferred over matched primer termini as substrates for pol κ as these would be encountered during the TLS extension step [82]. Pol κ can also generate single-base frame-shifts through template–primer misalignment wherein the wrong nucleotide is incorporated followed by a realignment of the primer on the template to loop out the mispaired base on the template (Figure 1) [47,81]. Taken together, these observations indicate a high tolerance of pol κ for noncanonical primer termini.

### 4.2. Pol Kappa Specificity and Cellular Functions

Benzo[a]pyrene. One of the most well-characterized bulky adducts, 10S-*trans*-*anti*-benzo[a]pyrene-7,8-dihydrodiol-9,10-epoxide (BPDE-*N^2^*-dG) (Figure 3), is derived from an environmental mutagen, B[a]P, present in tobacco smoke and combustion products of fossil fuel [65]. Replicative polymerases stall at BPDE-*N^2^*-dG, which is predominantly repaired through nucleotide excision repair (NER). However, if not repaired, the lesion leads to G to T transversion mutations in mammalian cells [83]. Pol κ catalyzes the insertion of a nucleotide opposite BPDE-*N^2^*-dG as well as the extension past this lesion with the highest efficiency and fidelity of all known DNA polymerases [14,16,51,52,53,54,55,84]. Other TLS polymerases bypass this lesion by misincorporation of dA [85,86,87,88]. The open catalytic core of pol κ easily accommodates the bulky BPDE-*N^2^*-dG adduct oriented towards the minor groove, which pairs with dC in a Watson–Crick base pair with an extra H-bond between *O^2^*-dC and *O^7^* from the BPDE-*N^2^*-dG to allow accurate insertion [23,89,90]. Comparable conformations of ternary complexes with and without adducted DNA templates have revealed unique interactions between the core and little finger domains mediated by the N-clasp domain, resulting in a partial coverage of the DNA duplex minor groove [89]. Moreover, both adducted and normal DNA substrates typically retain standard B-form, contributing to catalysis by pol κ. The 5ʹ orientation of the BPDE ring together with two stable water molecules provide coverage at the side of the minor groove in the active site and thus prevent formation of a wobble base pair and misinsertions during the extension step beyond the BPDE-*N^2^*-dG adduct [90]. Consequently, pol κ incorporates the next nucleotide after the lesion with higher fidelity than after a non-modified dG as observed in a primer extension assay [90].

Development of Nalm-6 human pre-B acute lymphoblastic leukemia cell lines (Nalm-6 cell lines), possessing high efficiency for gene targeting, has enabled control of pol κ expression to determine cell survival and the mutation spectrum after exposure to a mutagenic agent as well as to quantify mutation frequencies after replication and repair of a synthetic plasmid carrying a site-specific B[a]P lesion [91,92,93]. The mutation frequency of pol κ-deficient Nalm-6 cells treated with BPDE was increased in the *supF* gene forward mutation assays with a predominant mutation being that of dA [91]. Mutagenesis induced by BPDE was elevated in catalytically dead (CD) and knockout (KO) pol κ Nalm-6 cells as well as in mouse embryonic stem cells, confirming the catalytic role of pol κ in suppressing mutations by BPDE [6,11,13,16,92,94]. In mice with wild-type (*polk^+/+^*) or a catalytically inactive variant (*polk^−/−^*) of pol κ, mutagenesis induced by B[a]P was found to be insignificant in the colon, although spontaneous mutation frequency increased significantly in *polk^−/−^* mice at old age [95,96,97]. Thus, pol κ appears to catalyze TLS across endogenously generated DNA adducts caused by oxidative damage and alkylating agents in an error-free manner. Hakura et al. showed that B[a]P was not carcinogenic in mice when delivered alone [96]. Mice exposed simultaneously to B[a]P and dextran sulfate sodium (DSS), which causes inflammation in the colon, developed tumors. However, no significant difference was identified in the number of mice with tumors or tumors per mouse between *polk^+/+^* and *polk^−/−^* mice. Lipid peroxidation-induced adducts such as 1,*N^6^*-etheno-2′-deoxyadenosine, 8-hydroxypropano-2′-deoxyguanosine, and heptanone-etheno-2′-deoxycytidine were detected, but the BPDE-dG adduct was not. Thus, pol κ suppresses mutagenicity triggered by inflammation in the colon and lung of mice.

Phenylalanine 171 (F171) in the palm domain has been recognized as a biologically relevant non-catalytic residue via its interactions with the BPDE-*N^2^*-dG [54,89,90,91]. Replacing phenylalanine with an amino acid having a smaller side chain (F171A) resulted in more efficient bypass of BPDE-*N^2^*-dG in primer extension assays, suggesting that F171 inhibits pol κ activity [54]. Human cells expressing the F171A variant exhibited a significantly lower mutation frequency compared to cells expressing wild-type pol κ, further supporting the notion that interactions between F171 and the BPDE ring are energetically unfavorable [91]. Crystal structures show the phenyl ring of F171 in an altered conformation, moving away from the active site to accommodate the BPDE ring in the insertion stage complex [89,90].

Crosslinks. Special attention has been dedicated to the platinum-based anticancer drug cisplatin (CP) and the antitumor drug and antibiotic mitomycin C (MMC), of which the reactive species covalently bind DNA via formation of intrastrand adducts involving adjacent dGdG, dAdG, and nonadjacent bases dGdNdG and interstrand crosslinks (ICL) between two guanines in dGdC and dCdG sequences targeted by cisplatin and mitomycin C, respectively (Figure 3) [98,99,100]. ICLs represent the smallest part of the total CP- and MMC-induced modifications in a cell. Nevertheless, this type of DNA lesion is the most carcinogenic because the separation of leading and lagging strands during DNA replication results preferentially in double-strand breaks (DSBs), which can induce apoptosis [101]. Intrastrand adducts are removed mostly through the NER pathway while a complex mechanism using components of NER, homologous recombination (HR), and TLS pathways is required for ICLs repair [102,103,104].

Biochemical experiments have shown that the intrastrand adduct induced by cisplatin (3′-dG-Pt-dG-5′) substantially inhibits pol κ, which otherwise inserts a correct nucleotide easily opposite a native template at the first insertion step at 3′-dG [42,71,105]. However, the subsequent insertion and extension steps at dG-5′ are catalyzed with increased efficiency comparable to normal DNA replication. Pol κ is the most accurate relative to polymerases η and ι and predominantly incorporates correct nucleotides in insertion and extension steps [23]. Furthermore, pol κ can carry out strand displacement synthesis and can fully extend the primer having inserted a dNTP opposite cisplatin-interstrand crosslinked guanine [106]. Pol κ activity was characterized by low fidelity and high extension efficiency of interstrand-crosslink bypass, which depends on the conformation of the DNA duplex and the linker length [106].

Crystallographic studies have revealed a structural basis for human pol κ to bypass cisplatin intrastrand lesions as an efficient and accurate extender [23]. The analysis has suggested that pol κ accommodates lesions without a significant conformational change of its active site. Decreased efficiency could be due to deviation of the DNA damaged template from its protein-free conformation, resulting in a steric clash between the adduct and protein residues. Extensive interactions observed between the DNA template and the N-clasp, fingers, and little finger domains stabilize the ternary complex in a catalytically active conformation and thus orient nascent nucleotides for proper Watson–Crick base pairing.

Involvement of pol κ in the replication of DNA modified by CP was revealed in a study with human cells [107]. By using plasmids carrying a specific single-site lesion and knocking down TLS polymerases, pol κ activity in combination with pol ζ resulted in error-prone bypass past CP adducts. Research focused on the regulation of pol κ function in eukaryotic cells demonstrated the involvement of pol κ in DNA repair pathways induced by CP and MMC treatment [108,109,110]. TLS across ICLs formed on oligodeoxynucleotides with acrolein-derived crosslinks and on plasmids with trimethylene crosslinks demonstrated that human pol κ carried out accurate incorporation opposite the crosslinked guanine and extended the primer beyond the lesion [111]. Cell survival was adversely affected in pol κ-depleted human cells and mice following mitomycin C exposure, thus confirming the biological role of pol κ in tolerating the *N^2^*-*N^2^*-guanine ICLs in the minor groove [93,97,111]. Takeiri proposed a model mechanism of pol κ recruitment to the intrastrand adducts in vivo, wherein active pol κ suppresses point mutations by error-free DNA synthesis and prevents the formation of DSBs. In the absence of active pol κ, other TLS enzymes are recruited, resulting in higher mutation frequencies observed in mice with inactive pol κ. A similar model has been proposed for the role of pol κ in MMC-induced interstrand crosslinks in human cells based on findings from cytotoxic and genotoxic responses to MMC, mutation spectrum, and chromosome analysis [93].

Pol κ is an efficient DNA polymerase on templates with other lesions exhibiting cytotoxic, mutagenetic, and carcinogenic properties caused by exogenous and endogenous alkylating agents (e.g., S-adenosylmethionine) [67]. Many anticancer drugs (e.g., methyl methanesulfonate, MMS) have been developed as methylating agents because of their ability to trigger cell apoptosis [101,112,113]. The most prevalent and biologically relevant alkylated nucleobases are N3-methyladenine (N3-Met-dA), N7-methylguanine (N7-Met-dG), and *O^6^*-methylguanine (*O^6^*-Met-dG) (Figure 3) [114]. Base excision repair (BER) is the major pathway in the elimination of N-alkylpurines yielding an apurinic site (AP) as a byproduct, while *O^6^*-Met-dG is directly reversed by the action of *O^6^*-methylguanine DNA methyltransferase [115,116,117]. Moreover, methylpurine bases are unstable adducts in basic conditions undergoing either spontaneous depurination or imidazole ring opening to generate 5-*N*-methyl-2,6-diamino-4-hydroxyformamidopyrimidine (Met-FaPy) giving rise to G to T transversion mutations [118,119]. Overexposure to alkylating agents results in clustered abasic DNA lesions, defined as two or more damaged sites within 1.5 helical turns [120,121,122]. The repair mechanism of clustered lesions is a complex cellular process with escalated occurrence of deleterious double-strand breaks.

The initial evidence of pol κ involvement in TLS of the N3-Met-dA lesion comes from in vivo studies due to complications accompanying chemical synthesis of modified nucleotides. Analysis from survival assays on mice, chicken, and yeast cells missing essential repair proteins indicated that pol κ enhanced MMS resistance of Rev1-, Rev3-, and Rad30-deficient cells [123,124]. Additional studies supported a hypothesis that pol κ accomplishes its replication activity of methylated adducts in coordination with the Rev3 and Rev1/pol ζ pathways [125,126]. Primer extension experiments using a DNA template with 3-deaza-3-Met-dA (a stable analog of N3-methyl adduct) demonstrated that pol κ is moderately accurate and more efficient in the insertion of dT opposite undamaged dA than opposite N3-Met-dA [127,128]. Overall translesion synthesis decreased in human cells with pol κ removed by siRNA knockdown to a similar extent measured in cells depleted of both pol κ and pol ι, suggesting that pol κ plays an active role in extension beyond adducted DNA replicated by pol ι [128]. Human pol κ follows the A-rule while replicating a DNA template with an abasic site and requires dT as the next template base for further efficient extension [17,129,130,131].

## 5. Roles of Pol κ Beyond Translesion Synthesis

In addition to lesion bypass, pol κ contributes to other aspects of DNA metabolism. Human pol κ has roles in microsatellite stability [132,133] and replication of non-B DNA structures, including common fragile sites, G4 structures, and others [134,135,136]. In mouse cells, pol κ contributes to repair synthesis during nucleotide excision repair (NER) of UV-induced damage [137]. In human cells, pol κ also contributes to repair synthesis in NER and is recruited to repair sites by interactions with ubiquitylated PCNA via the pol κ UBZ domain [138].

Human pol κ colocalizes with PCNA in the nucleus, which increases with replication stress induced by treatment with hydroxyurea, UV, cis-Pt [139], or B[a]P [140]. The E3 ubiquitin ligase Rad18 is responsible for B[a]P-induced PCNA ubiquitylation and recruitment of pol κ [140]. Analogously, loss of the deubiquitinase USP1, which removes ubiquitin from PCNA, leads to pol κ-dependent genomic instability and reduction of replication fork speed, likely because of inappropriate engagement of pol κ in DNA replication [39]. Furthermore, pol κ is implicated in the Chk1 checkpoint response and recovery of replication from the stress of limiting dNTPs; pol κ interacts with the 9-1-1 checkpoint clamp as part of this response [141]. Another report indicates that pol κ has no effect on Chk1 activation but does play a role in replication fork restart under conditions of limiting dNTPs, dependent on the Fanconi Anemia pathway [142]. Taken together, these studies implicate pol κ in DNA metabolism beyond translesion synthesis.

## 6. Cancer-Associated Single-Nucleotide Polymorphisms (SNPs) in Human Pol κ

Numerous cancer-associated SNP variants have been identified in the *POLK* gene, which could impact pol κ function in several different ways. Mutations that decrease the activity of pol κ would render cells more sensitive to DNA damaging agents, including some chemotherapeutics. Mutations that alter the fidelity of pol κ will modulate the genomic (in)stability of tumors. Pol κ SNP variants with improved lesion bypass capability could play a role in resistance to DNA-damaging chemotherapy. The extension activity of pol κ can lead to mutations being fixed in the genome, and thus, increased primer extension activity can increase the mutation load if pol κ extends mismatched primer termini. Characterized pol κ mutations that have been reported in the literature are summarized in Table 1. Notably, the SNP variants associated with early-onset prostate cancer exhibit decreased primer extension activity in biochemical assays [143]. Some of the known SNPs modulate chemotherapy responses and cancer risk [144,145], suggesting the potential utility of drugs targeting pol κ [146].

## 7. Inhibitors

Pol κ is an attractive target for cancer therapies because it has been correlated with a shorter survival time in patients with glioblastoma as well as poor chemotherapy responses in some cases [92,144,145]. A high-throughput method to characterize the mechanism and inhibition of Y-family polymerases was developed through the use of a real-time fluorescent reporter [151]. This method can be used to monitor the inhibition of the polymerases by a small molecule in the presence of damaged template or damaged nucleotides [151]. The method was used to screen a library of ~16,000 bioactive molecules; the top 60 hits were then validated by primer extension with undamaged DNA [152]. From the top 60 hits, three compounds were studied further due to their specificity toward pol κ and one compound was observed to reduce XP-V cell resistance to UV light, which suggested in-cell inhibition of pol κ [152]. Another of the three compounds, MK886, was further studied through docking experiments and enzyme inhibition assays to determine its mechanism of action [153]. The results of these studies indicated that the mechanism by which the small molecule MK886 inhibits pols ι, η, and κ is different because the molecule can bind to different pockets identified through docking [153]. However, the small molecule results suggest that derivatization can lead to more potent and specific inhibitors, especially of pol κ. Recently an indole-aminoguanidine (IAG) scaffold-based inhibitor was identified to inhibit pol κ [75]. Mass spectrometry analysis of chemical footprinting reactions with an arginine-reactive probe localized binding sites for IAG-derived molecules in the fingers and little finger domains and in the N-clasp. Thus, it was concluded that the inhibitor disrupts interactions among these domains and alters normal N-clasp positioning, causing inhibition of pol κ activity [75]. Because the N-clasp is unique to pol κ and this molecule targets this domain, the molecule is attractive for further study, especially in chemotherapy-resistant cancers [75].

## 8. Conclusions

DNA pol κ plays a multitude of roles in DNA metabolism. Because pol κ is implicated in both cancer development and chemotherapy resistance, understanding the activity and specificity of pol κ, its SNPs, and the resulting variants can lead to new methods of detection and personalized medicine using inhibitors that target pol κ specifically. In the twenty years since pol κ was first discovered, many distinguishing characteristics have been identified, including its structure and unique domains, its preferred substrates, and disease-related mutations and patient outcomes. However, there are still several outstanding questions that can help guide the pursuit of understanding. Most biochemical work to date has been carried out with the polymerase core of pol κ; do other regions of the protein play roles in lesion specificity or extension activity? More studies with full-length pol κ need to be carried out in order to determine the contributions of less-characterized regions to the activity of pol κ. In addition, the effects on pol κ activity of the interactions of pol κ with its protein partners, including PCNA, Rev1, and others, should be probed in greater depth.

It is suggested that pol κ has a major role as an extender in TLS; what are the relative contributions to insertion or extension, and is this specific to each lesion? The search for specific inhibitors of pol κ and related SNPs is currently being pursued, especially ones that work in the cellular environment; is it possible to target specific SNPs? Addressing these questions will be important in understanding the basic biochemistry of pol κ as well as realizing the potential to improve cancer treatments.

## Figures and Tables

**Table 1:** *POLK* single nucleotide polymorphisms.

AA	ID	Effects	Domain	Tumor Site	References
L21F	rs3104729	30-fold decrease in incorporation opposite *N*^2^-CH_2_-(9-anthracenyl)-dG (*N^2^*-CH_2_-Anth-dG)	N-clasp	Prostate	[147,148]
E29K		Decreased insertion opposite abasic site (2–20×)	N-clasp	Prostate, early onset	[143]
I39T	rs3094258	Similar activity to WT with several types of DNA damage	N-clasp	Prostate, Melanoma	[147,148]
T44M		Lesion-specific reduction in activity; reduced activity with *N^2^*-CH_2_-Anth-dG, *O*^6^-Me-dG and abasic sites	N-clasp		[149]
S137S		Synonymous	Fingers	Prostate	[143]
G154E	COSM3856305	Decreased activity opposite model abasic site; pathogenic	Fingers	Prostate early onset, stomach	[143]
F155S		Decreased activity on model abasic site	Fingers	Prostate	[143]
P169T	rs148385845	Slight decrease in activity on undamaged DNA	Fingers	Lung ^a^	[148]
F171F		Synonymous	Palm	Prostate	[143]
D189G	rs111689950	Impaired for extension step of TLS	Palm		[147]
F192C	rs150515841	Slight increase in activity with *N*^2^-furfuryl-dG-containing templates	Palm		[148]
T205I			Palm	Prostate, early onset	[143]
R219I	rs3104717	Slight decrease in activity	Palm	Prostate	[147,148]
R219X	rs3094265 ^b^	Inactive	Palm		[147]
R246X	COSM3073601	5-10-fold less active with 8-oxo-dG-, *N^2^*-CH_2_-Anth-dG-, *O^6^*-Me-dG- and abasic-containing templates		Stomach	[149]
E292K	rs142203892	Similar activity as WT	Palm		[148]
R298H	rs151251843	Less active than WT on several different lesions	Palm	Large intestine ^a^	[148,149]
A329A	rs3213801	Synonymous, not meaningfully associated with breast cancer risk; more likely to respond to Pt-based chemotherapy	Palm	Breast	[144,145]
E419G	rs111584802	20-fold decrease in *k*_cat_*/K*_m_ on dG and 670-fold decrease on *N^2^*-CH_2_-Anth-dG, extension defect	Little finger ^c^		[147]
E419E		Synonymous	Little finger	Prostate	[143]
S423R	rs35257416 COSM6752124	1.6-fold more efficient than WT, 2-fold increased DNA binding affinity, pathogenic	Little finger	Melanoma, large intestine	[147,148]
A428A	COSM1070129	Synonymous	Little finger	Endometrium, Prostate	[143]
E430K			Little finger	Prostate	[143]
E430G		Low activity on AP site	Little finger	Prostate	[143]
Y432S	rs77612491	Less active on undamaged and damaged DNA, extension defect, decreased DNA binding affinity	Little finger	Melanoma	[147,148]
L442F		Low activity on AP site	Little finger	Prostate, early onset	[143]
Q447Q		Synonymous	Little finger	Prostate	[143]
E449K		Low activity on AP site, low fidelity	Little finger	Prostate	[143]
K461E			Little finger	Prostate	[143]
A471V	rs149894654	Moderate decrease in activity	Little finger		[149]
T473A	rs186798689	Decreased activity on undamaged and damaged DNA	Little finger		[149]
I487T			Little finger	Prostate	[143]
R512W		Decreased activity on undamaged and damaged DNA	Little finger		[149]
S528N				Prostate	[143]
D551N				Prostate	[143]
K564K		Synonymous		Prostate	[143]
D581N				Prostate	[143]
S678F				Prostate	[143]
L731F				Prostate	[143]
P861P				Prostate	[143]
D866E				Prostate	[143]
intron	rs10077427	Contributes to breast cancer risk, more likely to have progesterone receptor-positive tumors; Decreased progression-free survival with Pt-based chemotherapy		Breast	[144,145]
intron	rs5744533	Contributes to breast cancer risk, protective in postmenopausal women, no correlation with clinical phenotypes; More likely to respond to Pt-based chemotherapy		Breast	[144,145]
intron	rs3756558			Breast	[150]

^a^ 1000 genomes data; ^b^ Withdrawn from ClinVar in 2015; ^c^ In eukaryotic Y-family polymerases, the little finger domain is also referred to as the Polymerase-Associated Domain (PAD).
